# Tryptophan 375 stabilizes the outer-domain core of gp120 for HIV vaccine immunogen design

**DOI:** 10.1016/j.vaccine.2017.04.054

**Published:** 2017-05-25

**Authors:** Duoyi Hu, Dane Bowder, Wenzhong Wei, Jesse Thompson, Mark A. Wilson, Shi-Hua Xiang

**Affiliations:** a Nebraska Center for Virology, University of Nebraska-Lincoln, Lincoln, NE 68583, United States; b School of Veterinary Medicine and Biomedical Sciences, University of Nebraska-Lincoln, Lincoln, NE 68583, United States; c School of Biological Sciences, University of Nebraska-Lincoln, Lincoln, NE 68583, United States; d Department of Biochemistry and Redox Biology Center, University of Nebraska-Lincoln, Lincoln, NE 68583, United States

**Keywords:** Outer domain (OD), Gp120, HIV-1 subtype C, 375W, Structure-based design, Immunogenicity, Vaccine

## Abstract

The outer-domain core of gp120 may serve as a better HIV vaccine immunogen than the full-length gp120 because of its greater stability and immunogenicity. In our previous report, we introduced two disulfide bonds to the outer-domain core of gp120 to fix its conformation into a CD4-bound state, which resulted in a significant increase in its immunogenicity when compared to the wild-type outer-domain core. In this report, to further improve the immunogenicity of the outer-domain core based immunogen, we have introduced a Tryptophan residue at gp120 amino acid sequence position 375 (375S/W). Our data from immunized guinea pigs indeed shows a striking increase in the immune response due to this stabilized core outer-domain. Therefore, we conclude that the addition of 375W to the outer-domain core of gp120 further stabilizes the structure of immunogen and increases the immunogenicity.

## Highlights

• VRC01 epitope focused structure-based immunogen design.
• Gp120 outer-domain core was further stabilized by 375 tryptophan substitution.
• Epitope specific antibodies were predominately induced through guinea pig immunizations.

## 1 Introduction

The CD4-binding site (CD4-BS) of gp120 in HIV-1 has been recognized to be the most vulnerable site for antibody targeting and viral neutralization [1], [2], [3], [4], [5], [6], [7], [8]. Recently, identification of several potent neutralizing antibodies through the screening of HIV-1 patient sera, such as VRC01 [9] and PGV04 [5], have been known to be elicited by the CD4-BS of gp120. Further analysis of these antibodies has found that the binding sites for some CD4-BS antibodies primarily depend on the outer domain of gp120, which are unlike CD4 binding wherein the binding requires all the three domains: outer domain, inner domain and the bridging sheet [10]. It is known that the outer domain is relatively more stable than the inner domain, and the outer-domain core structure, which does not include the V3 loop, is even more stable [11], [12]. An optimized outer domain from a HIV-1 subtype A strain can bind to the CD4-BS antibodies VRC01 and PG04. The outer domain structure in complex with PG04 has been also solved [13]. The outer domain used as an immunogen has been tested for its immunogenicity previously, and it shows a comparable immune response to gp120, but a weaker response than the gp140 envelope trimer. However, it is an un-stabilized wildtype outer domain in which the V3-loop and the glycan site (antibody 2G12) were focused, not the CD4-BS [14]. A membrane anchored-outer domain is recognized by antibody b12 and is also found to have elicited CD4-BS antibodies [15]. All of these previous studies are based on the wildtype outer-domains, but the outcomes have provided the feasibility for developing outer-domain based immunogens.

CD4 binding to gp120 causes a large scale conformational change of gp120 which involves the gp120 core structure and the transitions of inner-domain layers (layer 1, 2 and 3), but also involves the movement of the major loops (V1, V2 and V3) [16], [17], [18], [19]. For instance, the V2-loop joins the bridging sheet formation with the C4 region β20-β21 hairpin of the outer-domain, and the V3-loop opens up to contact the coreceptor, CCR5 or CXCR4 [17], [18], [20]. A tryptophan residue at amino acid position 375 (375W) of gp120 will fill the Phenylaniane-43 (Phe-43) cavity and stabilizes the gp120 into a CD4-bound conformation, but does not completely achieve the CD4-bound state [21], [22], [23]. More stabilization changes such as the addition of disulfides, salt-bridges added to the S375W conformation of the gp120 core structure have enhanced the stability and increased the immunogenicity [22], [24]. However, in respect to the outer-domain core structure only, we do not know whether the 375W can play a role in further improving the stability of the CD4-bound state. Here we have conducted the research into an outer-domain based HIV immunogen design. The antigenic nature of the CD4-BS has been recognized as conformation dependent because it is known to be located at the junction of three domains. Interestingly, the binding sites of some CD4-BS antibodies such as VRC01 or PGV04 are actually located on the outer domain, and the binding is almost independent from the inner domain and the bridging sheet. Since the outer-domain of gp120 is a relatively stable domain, it has become an ideal antibody target for vaccine immunogen design.

In our previous study, a stabilized outer-domain, through the introduction of two-disulfide bonds, was significantly more immunogenic than the wild-type outer domain. To further improve this outer-domain based immunogen, we have introduced a Tryptophan (W) mutation at the position 375, which is known to be the Phe-43 cavity filling residue in the CD4-BS. We expect that the 375W will further stabilize the outer-domain into the CD4-bound conformation in addition to the two-disulfide bond stabilization. Thus, it will serve as a better immunogen to induce the production of CD4-BS neutralizing antibodies.

## 2 Materials and methods

### 2.1 Outer domain OD3 mutagenesis

The outer domain mutant 3 (OD3) of HIV-1 gp120 from subtype C strain 1084i essentially was designed based on the outer domain mutant 2 (OD2) structure as described previously [25]. The S375W mutation was introduced by Site-directed mutagenesis using the mutagenesis kit from Agilent. The sequences of the mutations were confirmed by DNA sequencing. The models of the OD1, OD2 and OD3 structures were predicted based on the gp120-VRC01 structure complex (PDB: 3NGB) from an HIV-1 subtype B strain, and conducted using Discovery Studio Client 4.0 (Biovia, San Diego, CA). The protein sequence numbering of OD1, OD2 and OD3 is based on the envelope sequence of the HIV-1 prototypic HXBc2 strain [26].

### 2.2 Cloning and expression of OD1, OD2 and OD3

The molecular clones of OD1 and OD2 were made during previous work [25], and the OD3 mutant was generated by mutagenesis in the vector pET28b (Novagen). To express the recombinant proteins in *E. coli BL21 (DE3) pLysS cells* (Invitrogen)*,* the pET28b-OD1, OD2 or OD3 plasmids were transformed into the cells and a single colony was grown overnight in LB media supplemented with kanamycin (50 mg/L) at 37 °C until the optical density (OD) reached between 0.4 and 0.6 at 600 nm. The cells were then induced by addition of isopropyl-D-thiogalactopyranoside (IPTG) at a final concentration of 1 mM and were further incubated for 4 h at 37 °C. The cells were then harvested by centrifugation at 5000*g* for 10 min, washed once with STE buffer (100 mM NaCl, 10 mM Tris, and 1 mM EDTA, pH 7.5) and finally resuspended in STE buffer containing 0.5% NP40, lysozyme (100 mg/L) and supplemented with protease inhibitors. After incubation for 30 min at 4 °C, the resuspended cells were disrupted by sonication and the lysate was centrifuged (12,000*g*, 10 min, 4 °C) to separate the insolubilized fraction. Both the supernatant and the insolubilized pellet fraction were tested for the presence of recombinant proteins by running SDS-PAGE followed by Coomassie blue staining. The majority of the expressed protein was detected in the insolubilized fraction of inclusion bodies. The insoluble fraction was further purified by sequentially washing with 1x PBS containing 2 M urea, 0.1% SDS, 1% NP-40 and 1% TritonX-100, and was solubilized in solubilization buffer (50 mM NaCl, 25 mM Tris (pH-8.0), 2 mM EDTA, 8 M Urea and 5 mM DTT). The protein was finally renatured by successive dialysis against refolding buffer (50 mM Tris, pH 8.0, 400 mM l-Arginine, 2 mM EDTA, 10 mM DTT and 10% glycerol) containing 4, 2, 1 and 0 M Urea at 4 °C. Ni-NTA resin beads (Thermo Scientific) were used to further purify the protein. The concentrations of the protein samples were determined by a BCA protein kit (Pierce). The protein samples were finally evaluated by Coomassie blue staining and Western blotting.

### 2.3 Western blot assay

Purified protein samples were loaded onto a 10% SDS-denaturing gel along with negative (bovine serum albumin (BSA)) or positive (recombinant gp120) controls. The gels were run for 75 min at 60 V. Nitrocellulose membrane transfer followed SDS-PAGE. After 30 min of blocking with 5% non-fat dry milk in phosphate-buffered saline (PBS), nitrocellulose membranes were incubated for one hour at room temperature with primary antibodies. Following six washing steps (five minutes each) with PBS + 0.1% Tween-20, horseradish peroxidase (HRP) conjugated secondary antibodies were incubated with membranes for 30 min at room temperature. Six washes were followed as previously described. The outcome of antigen-antibody binding was detected either using SuperSignal West Dura Extended Duration Substrate kit (Pierce) or using a BioRad ChemiDoc.

### 2.4 Circular Dichroism (CD)

A desalting column (zeba™ Spin Desalting Columns, 7 K MWCO, 5 mL) was used to remove l-arginine from the protein solution to avoid noisy signals. A Jasco J-815 CD spectrophotometer equipped with Peltier-type thermostat in the cuvette holder and a water circulator was used to collect the data. The acquisition and data analysis software are also from Jasco. Data were recorded using a 0.01 cm pathlength, 1500 µL circular quartz cuvette at 20 °C. The data were exported in XY asci format and analyzed with the program CD-Pro located at the Dichro Web-site from the University of London, for the purpose of estimation of the secondary structure of the protein. The data were converted to molar ellipticity (deg·cm2·dmol-1) from raw machine units (millidegrees) with the same resolution as the acquired data (1 data point per nanometer). The cell path, average molecular weight and protein concentration were provided prior to the fitting, which was carried out using the various models available (CONTINLL and SELCON3 yielded the lowest RMSD values) until a satisfactory fit was obtained. The results were imported into Sigmaplot (SPSS), which was used to plot the data along with the simulated secondary structure spectrum. The three protein samples were measured at the same concentration of 0.35 µg/ml, solubilized in buffer containing 20mmTris-HCl and 2 mm EDTA at pH 8.0, and the recording path-length is 0.01 cm.

### 2.5 Guinea pig immunizations

The standard 63-Day immunization protocol of Cocalico Biologicals, Inc. (CBI) for guinea pigs was followed. 16 guinea pigs were assigned evenly into 4 groups: inoculated with OD1, OD2, OD3 or adjuvant only (Complete Freund’s Adjuvant for initial inoculation, but Incomplete Freund’s Adjuvant for boosters). Three booster inoculations by Subcutaneous (s.c.) injections were carried out following the first injection. Blood samples were collected from all guinea pigs on Day 0, prior to immunization as pre-bleed samples; on Day 35 as test bleed samples after 2 boosts on Day 14 and Day 21, respectively; and on Day 63 as exsanguination samples following the third boost on Day 49. Blood samples collected (pre-bleeds, test bleeds and final bleeds) were used in antibody testing, ELISA and neutralization assays. All animal experiments were performed according to the protocol approved by the IACUC of CBI (Reamstown, PA) (see Table 1).

**Table 1:** Immunization schedule.

Day	Procedure	Antigens and Adjuvant (CFA&IFA)^a^
0	Pre-bleed	
	Initial inoculation	200 µg antigen/animal with CFA
14	1^st^ Boost	100 µg antigen/animal with IFA
21	2^nd^ Boost	100 µg antigen/animal with IFA
35	Test bleed	
49	3^rd^ Boost	100 µg antigen/animal with IFA
63	Final bleed	

a CFA, Complete Freund’s Adjuvant; IFA, Incomplete Freund’s Adjuvant.

### 2.6 ELISA

Flat-bottom with low evaporation lid 96-well plates (Falcon™) were coated with 200 ng per well of the testing protein antigens (OD1, OD2 or OD3) diluted in coating buffer (15 mM Na_2_CO_3_ and 35 mM NaHCO_3_, pH 9.6). Prior to blocking, plates were washed with phosphate buffered saline with 0.1% Tween-20 (PBS-T, Sigma-Aldrich) 3 times. Plates were blocked with 200 μL/well blocking buffer (PBS-T with 5% milk) at room temperature for 1 h. After 3 washes with PBS-T, 100 μL/well of guinea pig sera diluted in PBS-T to different ratios was added and incubated at room temperature for 2 h. Plates were washed 5 times prior to the addition of 100 μL/well of HRP-conjugated goat anti-guinea pig IgG secondary antibody (Jackson ImmunoResearch Laboratories Inc.), and incubated at room temperature for 2 h. Plates were washed 5 times, and 100 μL/well SureBlue Reserve TM TMB substrate (KPL) was added and incubated for 1 min in the dark. 100 μL/well of 1 M HCl was added to stop the reaction. Absorbance data was collected at 450 nm using an ELX 800 UV universal microplate reader (Bio-Tek Instruments).

### 2.7 Production of pseudotyped HIV viruses

HIV-1 isolates used for neutralization assay were pseudotyped HIV-1 viruses produced by transfection of 293T cells with three plasmids: pHIV-1-luc, pCMV Gag-Pol, and pSVIIIenv of different isolates. Transfection of these three plasmid produces luciferase-expressing HIV-1 isolates in 293T cells after a three day incubation period. Transfected 293T cells were grown at 37 °C with 5% CO_2_, and maintained in DMEM medium with 584 mg/L l-Glutamine (Gibco), supplemented with 10% FBS and 1X penicillin-streptomycin. Twenty-four hours prior to transfection, 1.5 × 10^6^ cells were plated in 10 cm dishes. Cells were transfected by adding 6 µg of replication-deficient HIV-1 luciferase plasmid pHIV-1-luc, 2 µg HIV-1 envelope construct pSVIIIenv-YU2/1084i/89.6/JRFL/HXBc2/ADA, and 2 µg CMV backbone plasmid diluted in 1 mL unsupplemented DMEM. Polyethylenimine (PEI) was the transfection reagent used in these experiments. Cell supernatants were collected at 72 h post-transfection, and were purified through a 0.45 µM syringe tip filter. The amount of virus in the supernatants was measured by reverse transcriptase (RT) assay [27]. Viruses harvested from the supernatant of the culture media were stored at −80 °C.

### 2.8 Viral neutralizing assay

The viral neutralization assay was based on methods previously described [28], [29]. In brief, TZM-bl cells were incubated at 37 °C with 5% CO_2_, and maintained in DMEM medium with 4.5 g/L D-glucose and 584 mg/L l-Glutamine, supplemented with 10% FBS and 1X penicillin-streptomycin. One day prior to infection, cells were seeded at 1.5 × 10^4^ per well in flat-bottom, black-sided 96-well plates (Grenier Bio-One). In a separate 96-well plate, heat-inactivated guinea pig serum was serially diluted to a final volume of 150 μL cell growth media, mixed with 50 μL cell growth media containing 2500 RT units of pseudotyped HIV, and incubated for 1 h at 37 °C with 5% CO_2_. During this incubation, TZM-bl growth media was replaced with 50 μL media containing 160 μg/mL DEAE-Dextran (Sigma-Aldrich) (40 μg/mL final concentration). The serum-virus mixture was added to cells and incubated at 37 °C for 48 h. The luciferase assay system (Promega) was used to detect Tat-inducible luciferase; luminescence was measured on a Veritas Luminometer (Turner Biosystems). Percent neutralization was determined as described previously [25].

### 2.9 Statistical analyses

All data statistical analyses were performed using Graphpad Prism (v. 6.0). The statistical significances were determined by using the Holm-Sidak T-test method [30], [31]. To determine the statistical significance of the antibody titers between day 0 and day 35, or day 0 and day 63, statistical significance analysis was conducted with alpha = 5.000% (Fig.6A). To determine the statistical significance of the antibody titers between OD1 and Adjuvant, OD2 and Adjuvant, OD3 and Adjuvant, OD1 and OD3, and OD2 and OD3, the Holm-Sidak T-test method was used with alpha = 10.000% (Fig.6B). To determine the statistical significance of the virus-specific neutralizing activities between OD1 and Adjuvant, OD2 and Adjuvant, OD3 and Adjuvant, OD1 and OD3, and OD2 and OD3, the Holm-Sidak T-test method was used with alpha = 5.000% (Fig.7A).

## 3 Results

### 3.1 Structure-based design of the outer-domain into the CD4-bound conformation

**Fig. 1:**
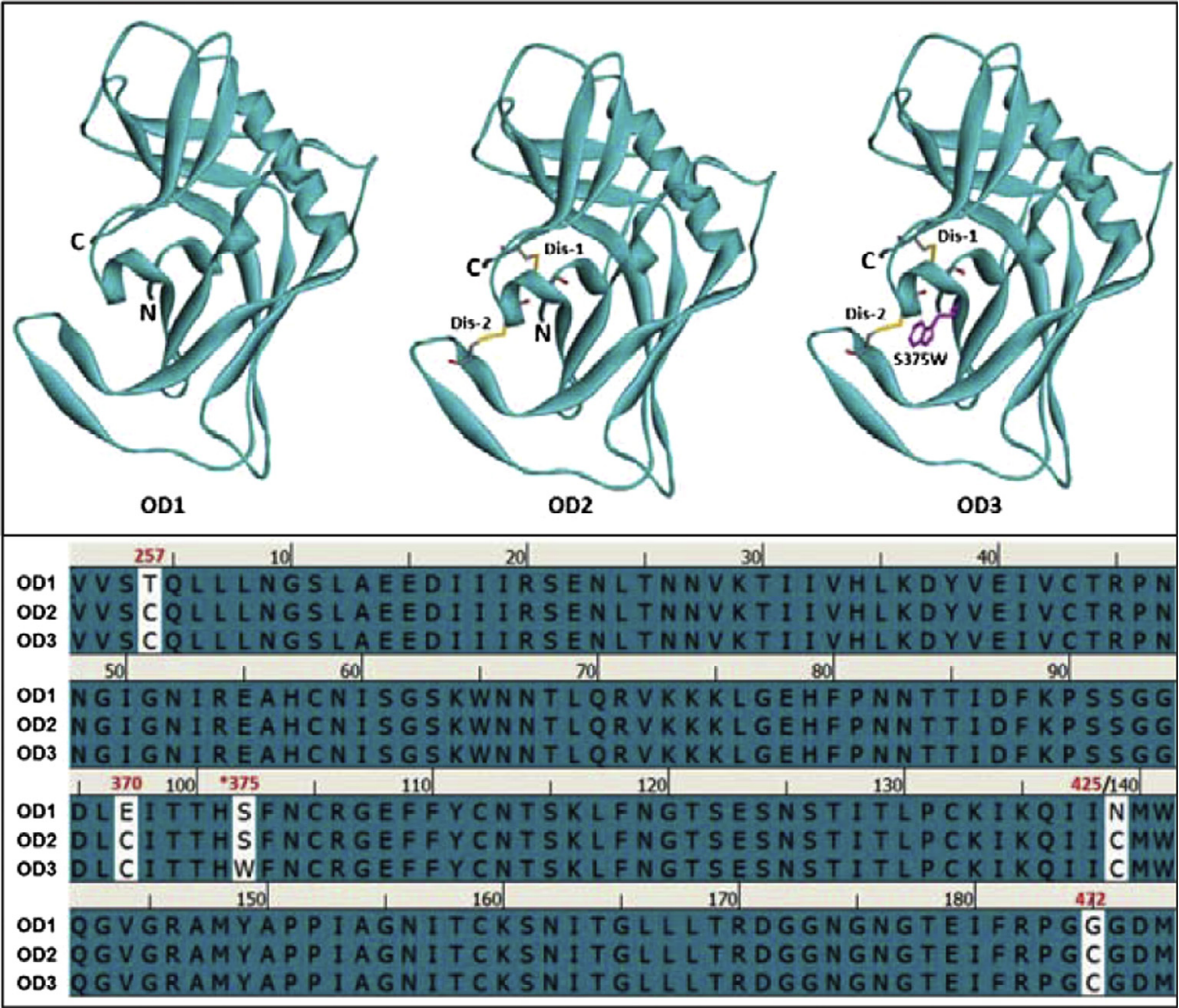
Models of the three designed immunogens (upper panel) and their amino acid sequence alignment (lower panel) are made using Discovery Studio and the sequence is from the envelope glycoprotein gp120 of HIV-1 subtype strain 1084i. The outer domain (OD) is cut-off from position 254 of the full length gp120 molecule, which corresponds to position 1 of the outer-domain numbering in this diagram. The protein sequence numbering is based on the full length sequence of gp120 standard numbering system [26], but sequence numbers the for the outer-domains only are also indicated in the sequence alignment and in the brackets for the mutations: Dis-1, disulfide bond-1, T/C257-G/C472 (T/C4-GC185); Dis-2, disulfide bond-2, E/C370-N/C425 (E/C97-N/C139); S375W (S102/W). OD1, wild-type outer domain; OD2, with two disulfide-bonds; OD3, with two-disulfide-bonds plus S375W mutation.

Based on the X-ray crystal structure of gp120 in the CD4-bound conformation (PDB: 3NGB) [11], we have separated the outer domain from the gp120 structure to design the immunogen. As indicated in our previous work, we introduced two-disulfide bonds to stabilize the outer-domain to maintain the CD4-bound conformation (OD2) to generate targeted CD4-BS antibodies. In this research, we have further improved the CD4-bound conformation of OD2 by adding an additional serine to tryptophan mutation at position 375 (S375W). The rationale for this design is based on the previous finding that 375W is a Phe-43 cavity filling residue in the gp120 structure which can stabilize the gp120 into the CD4-bound conformation [11], [21]. It was assumed that this 375W mutation would further improve the OD2 conformation into the CD4-bound state, which is the ideal conformation to be achieved for vaccine design against the CD4-BS. The wild-type outer domain (OD1), the two-disulfide stabilized outer-domain (OD2) and the added S375W substitution outer-domain (OD3) are all shown below in Fig. 1, including their structural models and the protein sequences. In this research, we have purified the outer domain proteins and compared them side-by-side through molecular characterization and immunization. As in our previous study on OD2, we have focused on an HIV-1 subtype C strain (1084i) that was originally isolated from Zambia [32]. The 3-dimensional structure model of 1084i gp120 was established during the previous study using a molecular modeling approach. One disulfide serves to stabilize the N- and C-termini of the outer domain as they are located closely to the antibody VRC01 binding site. The other disulfide was added to fix the β20/21 hairpin in the CD4-bound conformation, which is favorable for VRC01 antibody binding (Fig. 1). Because this design focused specifically on the VRC01 binding site in the core structure of gp120, the V1V2 loops along with the V3-loop were removed. It is anticipated that these modifications will make this immunogen more rigid as the CD4-bound conformation is supposed to elicit potent neutralizing antibodies like VRC01 and PG04.

### 3.2 Characterization of the outer domain immunogens

**Fig. 2:**
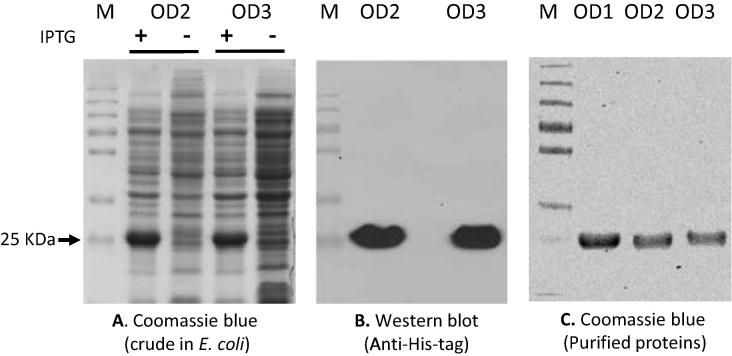
Protein expression and western blot verification. (A). Coomassie blue staining to evaluate the OD2 and OD3 protein expressed in *E. coli BL21 (DE3)* pLysS cells which were induced by 1 mM IPTG. (B). Western blot of OD2 and OD3 protein using anti-His tag antibody. (C). Purification of OD1, OD2 and OD3 protein immunogens from the Nickel column, and with the Coomassie blue staining. Note that the OD1 protein was purified from previous work in the laboratory.

**Fig. 3:**
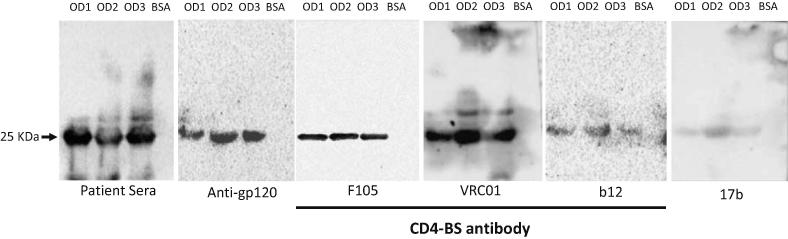
Characterization of the three immunogens by Western blotting. After SDS-PAGE and membrane transfer, protein samples of OD1, OD2, OD3 and BSA were probed with pooled serum from HIV-1 positive patients, an anti-gp120 polyclonal antibody, CD4-BS monoclonal antibodies VRC01, F105, b12, and a CD4-induced monoclonal antibody 17b.

The designed and synthesized immunogen genes OD1, OD2 and OD3 were cloned into the pET28b vector (Novagen) to express the proteins in *E. coli BL21 (DE3)* pLysS cells (Invitrogen). The proteins were characterized by SDS-PAGE, followed by Coomassie blue staining (Fig.2A). The induced protein bands were detected in the Coomassie gel as expected the molecular weight of 25 kDa, representing the outer domain proteins of HIV 1084i gp120, and were also confirmed by Western blot using an antibody against His-tag (Fig.2B). Large amounts of OD1, OD2 and OD3 protein were then prepared and purified via Ni-column for use in immunizations. After purification, the OD1, OD2 and OD3 proteins were further checked for the purity by SDS-PAGE using Coomassie blue staining (Fig.2C). Clear bands of OD1, OD2 and OD3 confirmed the high purity of these immunogen proteins. Some gp120-specific monoclonal antibodies were also used to test the immunogen conformation and recognition by CD4-BS antibodies. As expected, the CD4-BS antibody VRC01 showed strong affinity to OD1, OD2 and OD3, and the antibodies F105 and b12 also showed positive binding. The CD4-induced (CD4i) antibody 17b also bound to the immunogen proteins (Fig. 3). Note that some minor weak unspecific bands present in the patient sera and VRC01 gels in Fig. 3 are assumed to be gp120 dimers. The outer-domain glycan dependent antibody 2G12 binding was negative (data not shown) [25]. This is due to the lack of glycans on the proteins which were produced in prokaryotic cells [33], [34]. Although the VRC01 antibody, when bound to gp120, contacts the V5 loop, which is glycosylated, the glycan is located at the outside part of loop and is not actually involved in the binding [9].

**Fig. 4:**
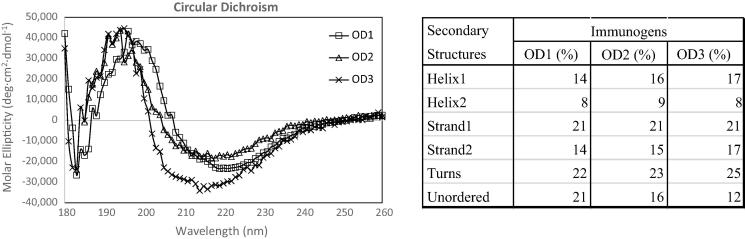
Circular dichroism (CD) spectra of three immunogens with representative secondary structures. (A). CD spectra of OD1, OD2, and OD3 were measured at pH 8.0 and at 20 °C and between 180 and 260 nm wavelengths. (B). The data was analyzed by software CDSSTR and is summarized in the table.

Further characterization of the folding and conformation of the OD1, OD2 and OD3 antigens was performed using Circular Dichroism (CD) spectrophotometry. The data are shown in Fig. 4. It can be seen obviously that the structural regions (α-helix, β-strand and the turns) were increased from OD1(79%), OD2(84%) to OD3(88%), and the unordered regions were reduced from OD1(21%), OD2(16%) to OD3(12%). Therefore, the CD spectra indicates that the OD3 has more ordered secondary structures than OD1 and even OD2. This suggests that the OD3, which has the 375W is more stabilized at the secondary structural level.

### 3.3 Immunogenicity test in guinea pigs

To evaluate the immunogenicity of OD3 and compare it to OD1 and OD2, we prepared the three antigens and immunized guinea pigs at the same time so that a side-by-side comparison could be achieved. The sample groups of OD1, OD2 and OD3 were injected with immunogen and the adjuvant, but the control group was only injected with the adjuvant and no immunogen. Antisera were collected from all immunized guinea pigs at Day 0 (pre-bleed), on Day 35 (test bleed) and on Day 63 (final bleed). The details of the guinea pig immunization are described in the methods section.

**Fig. 5:**
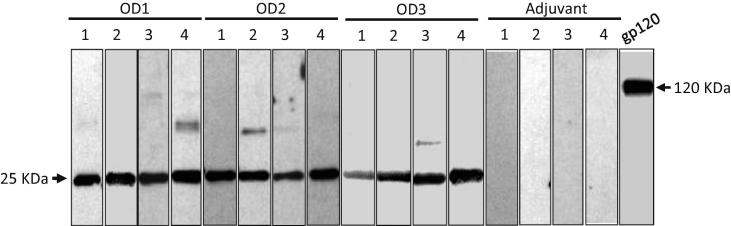
Western blotting analysis of antisera from immunized guinea pigs. Each serum from an individual animal in the test group was tested against its immunogen (OD1, OD2 or OD3). As designed, there were four animals in each group. All antigen-immunized groups showed antibody-binding activity to gp120, but here only one is shown as a positive control. All animals in the adjuvant only immunization group showed no antibody binding activity to OD1, OD2, OD3 and BSA (shown in Adjuvant 1, 2, 3, and 4 respectively).

The antisera from the animals were tested by Western blotting to check whether the outer domain immunogens raised specific antibodies to the immunized antigens. The results were positive and are shown in Fig. 5. All antisera from the animals of the OD1, OD2 and OD3 groups had antibodies against the corresponding antigens, but in the adjuvant group, all four animals were negative against the gp120 antigen (Fig. 5). The minor weaker unspecific bands in the Patient sera and VRC01 gels are assumed to be dimers or trimers.

**Fig. 6:**
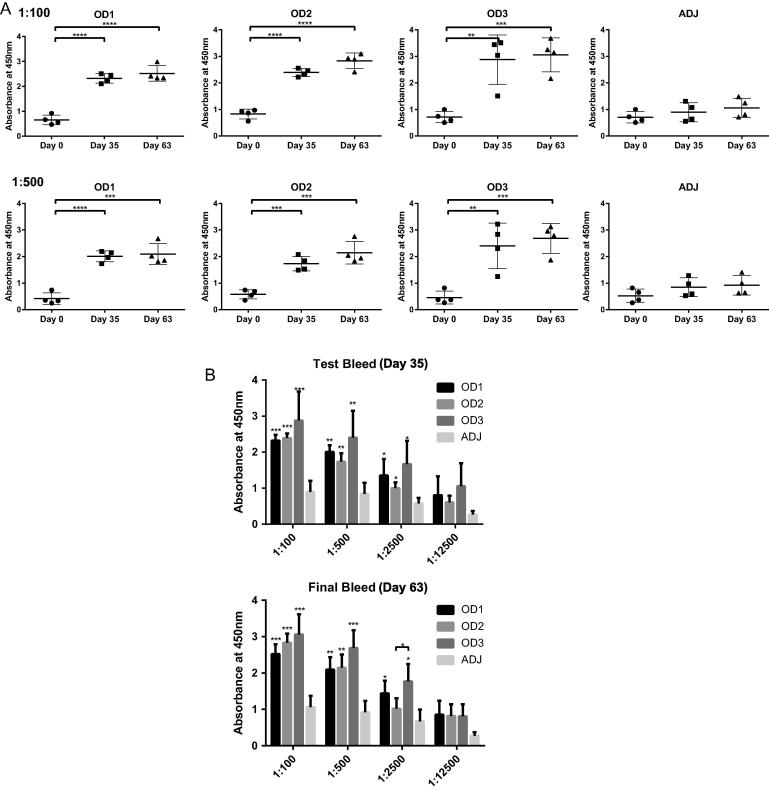
ELISA analysis of antibody titers (absorbance at 450 nm) in the antisera from immunized guinea pigs and their individual antigenic differences. (A). 96-well plates were coated with three antigens, then were probed with dilutions of pre-bleed serum (Day 0), test bleed serum (Day 35), or final bleed serum (Day 63). Experiments were performed in triplicate and figures represent an average data of multiple experiments. Upper four panels, serum dilution rate 1:100. Lower four panels, serum dilution rate 1:500. The pre-bleed, test bleed and final bleed antibody titers were compared in four groups (4 animals per group) immunized with OD1, OD2, OD3 or ADJ (adjuvant only). (B). ELISA analysis of antisera from immunized guinea pigs comparing both the antigenic differences among three immunogens with the control group ADJ, and the antigenic differences between OD1 and OD3 or OD2 and OD3. The sera from Day 35 (Test bleed group) and Day 63 (Final bleed group) were both tested.

The antibody titers in each animal were measured by Enzyme-linked immunosorbent assay (ELISA), and the data are shown in Fig.6A, (also see Fig. S1). It is clear that the antibody titers have significantly increased from the time post-immunization at Day 0 (pre-bleed) to Day 35 (test bleed) and Day 63 (final bleed). In general, there were not significant differences in antibody titers among the animals in each group, except for one animal in OD3 group which seemed to have responded poorly (Fig.6A). These antibody response data suggest that the outer-domain antigens induced the animals’ immune responses and elicited specific antibodies against these antigens. The average antibody titers from each immunization group showed that OD3 was higher than groups OD1 and OD2 on Day 35 or Day 63 (Fig.6B). This suggests that the OD3 immunogen was more immunogenic than OD1 and OD2, although one animal had a poorer response to the OD3 immunogen. The data shows that OD3 animals still showed better immune responses in general.

**Fig. 7:**
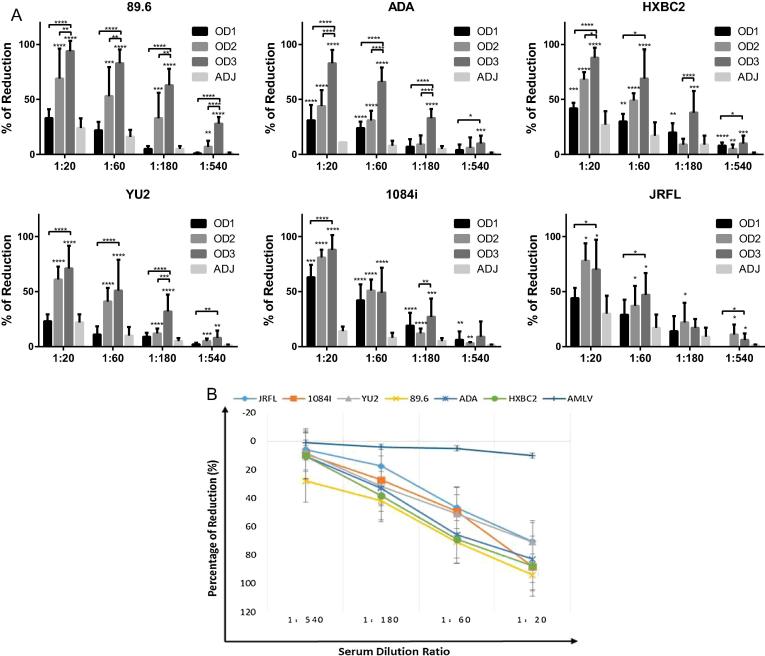
Virus neutralization assays of antisera from immunized guinea pigs and comparisons of the neutralizing activities of three immunogens (OD1, OD2 and OD3). (A). To compare the biological activity of the virus-specific neutralizing antibodies in guinea pig serum induced by the designed antigen OD3 with OD1, OD2 and ADJ, at different serum dilution rates. Day 63 final bleed serum were used in this study. Each panel represents their activities against 6 different HIV-1 isolates: 89.6 (dual tropic virus), ADA (R5 virus), HXBc2 (X4 virus), YU2 (X5 virus), 1084i (sub-type C R5 virus), JRFL (R5 virus). (B). Virus neutralization assay of antisera from OD3-immunized guinea pigs comparing serum neutralizing activities against diverse HIV-1 isolates and a non-HIV-1 isolate. This figure compares the neutralizing activity of OD3-immunized serum in six HIV-1 isolates, and one non-HIV-1 virus strain AMLV (Amphotropic Murine Leukemia Virus) serving as a negative control.

To further evaluate whether these antibodies produced by the animals have neutralizing activities against HIV infection, a panel of HIV-1 strains were tested which included a Tier 1 strain virus (HXBc2) and several Tier 2 strain viruses (89.6, ADA, YU2, JRFL, 1084i) [35]. The neutralization results evidently showed that the serum from animals who received the immunogen OD3 was more effective than OD2 or OD1, and the neutralization activity increased from Day 35 (test bleed) to Day 63 (final bleed) significantly (Fig.7A). More detailed virus neutralization data have been presented in supplemental Figs. S2 and S3. All these data suggest that more neutralizing antibodies were produced from the more stabilized immunogen OD3 at Day 35 and 63. If we summarize the immunization data from the final bleeds and compare their antibody titers (1:100) and the neutralization activities (1:60), we can conclude that the OD3 apparently is a better immunogen than OD2 and OD1 (Table 2). We can also see that in Table 2, the antibody titers did not significantly increase from OD1 to OD2 and OD3, but the virus neutralizing activities were largely increased, some of them even having a two, three or four times increase, as with the strains 89.6 and YU2. This implies that immunogen stabilization has increased the elicitation of specific neutralizing antibodies against HIV.

**Table 2:** Summary of OD antigen immunogenicity.

	Ab titer (%)	Neutralization (%, 1:60)					
	Final bleed (1:100)	89.6	ADA	HXBc2	YU2	1084i	JRFL
OD1(WT)	100	100	100	100	100	100	100
OD2(DS)	113	242	132	163	364	121	126
OD3(DS + 375S/W)	121	378	280	228	453	117	160

Finally, we examined whether these antibodies specifically neutralized HIV. A non-HIV, but similar retrovirus, AMLV (Amphotropic murine leukemia virus) was used as a control. The results showed that several HIV-1 strains, JRFL, YU2, ADA, HXBc2 and 89.6 and including the subtype C strain 1084i were neutralized by immunized guinea pig antisera, but AMLV was not neutralized even at a 1:20 dilution (Fig.7B). This suggests that the antibodies in the sera were specific against HIV viruses. The non-HIV-1 AMLV served as a negative control, reflecting that the neutralizing antibodies are HIV-1 specific since they did not neutralize AMLV. Among these HIV-1 isolates, the dual-tropic 89.6 seemed the most vulnerable strain to OD3-induced antibody neutralization, followed by the lab-adapted strain HXBc2.

## 4 Discussion

This research has shown the Phe-43 cavity filling residue 375W can further stabilize the outer-domain core structure of gp120 into the CD4-bound conformation in addition to the base of the stabilization facilitated by the two-disulfide bond. The stabilized outer-domain core clearly showed a significant improvement in the immune response through the immunization of guinea pigs. The neutralization activities against a variety of the HIV-1 isolates have shown the potency and broad coverage of the induced antibodies. We have noticed that the sera from OD3 immunization seem to have weaker neutralizing activity against the subtype C strain 1084i than other strains. We do not know why this autologous strain is more difficult to neutralize. Further investigations are needed to find the mechanisms, which are also useful for future antigen design.

This work again demonstrates that the CD4-BS of gp120 is an important region for antibody targeting, and the CD4-binding portion of the outer-domain appears to be even more imperative for antibody targeting. As has been documented, potent neutralizing antibodies such as VRC01 and PG04 are examples of targeting to this region. If we compare the whole gp120 to the outer-domain core as vaccine candidates, it is obvious that the outer-domain core has advantages in terms of molecular size, stability and epitope focusing. The outer-domain core structure avoids the unstable inner domain and the flexible loops which reduces the unwanted immunogenic face of the inner domain and focuses on the stable epitopes of VRC01-like antibodies. Therefore, we can see the outcomes of immunization data on the previous tested OD2 [18] and this research on OD3. It is possible that more specific neutralizing antibodies might be raised but less total antibodies were produced which suggests that non-neutralizing antibodies were reduced in the immunizations with OD2 and OD3. Based on this set of immunization data with OD1, OD2 and OD3, we can clearly see that the structure-based immunogen design has more rationale and accuracy for inducing specific neutralizing antibodies, which can be essentially explained by the epitope structural relationship between antigens and antibodies. We will continue to pursue this through investigations which purify the antibodies elicited from these immunizations. Then, we will examine their structures, binding epitopes and affinity, and compare to the known reference antibodies such as VRC01 and PG04. Thus, we may be able to gain more insights into the use of structure-based approaches to design more effective immunogens for HIV/AIDS vaccine development.

## 5 Conclusions

From these outer-domain structure-based immunogens, we can conclude that the stabilized outer-domain in a CD4-bound conformation elicits a better immune response than the un-stabilized conformation. The stabilized outer-domain core could serve as a better vaccine candidate in HIV vaccine development. In terms of approach, the structure-based immunogen design has a rational advantage and is demonstrated to be a good approach in this field.
