# Supplementary figures and images for: Tryptophan 375 stabilizes the outer-domain core of gp120 for HIV vaccine immunogen design
Source: Vaccine. 2017 May 25;35(23):3067–75. doi: 10.1016/j.vaccine.2017.04.054 (PMC5440730; doi:10.1016/j.vaccine.2017.04.054)

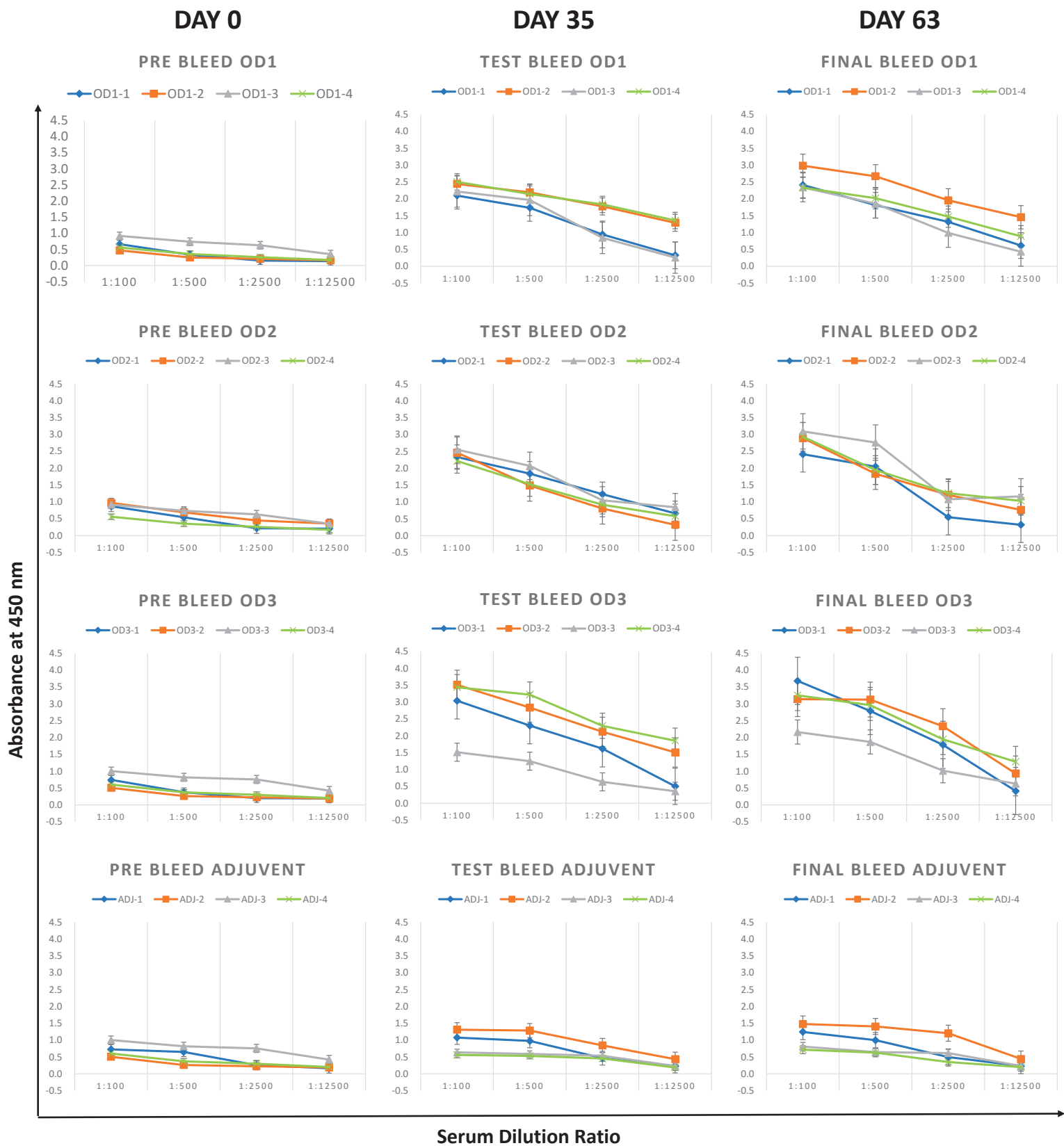

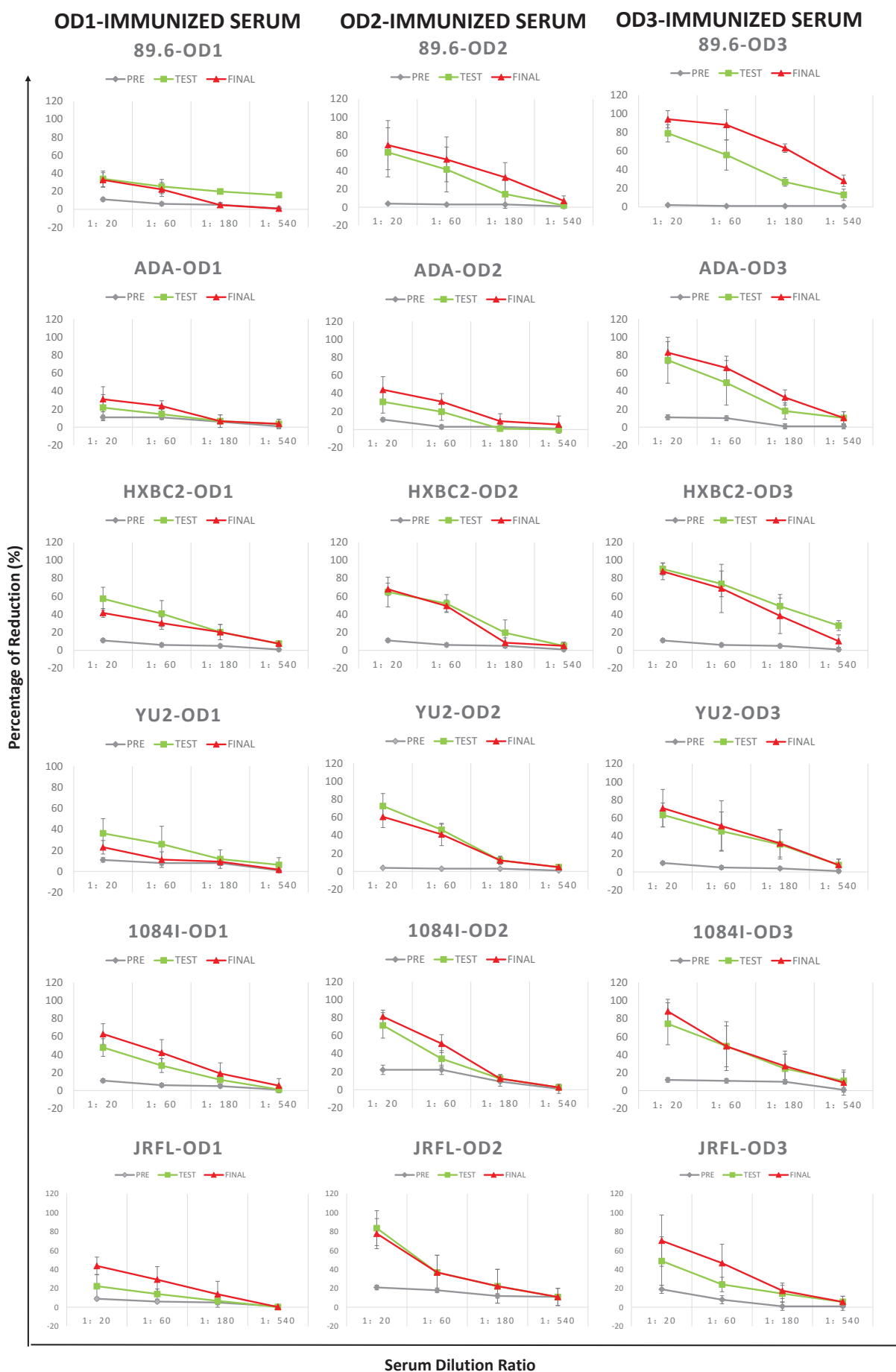

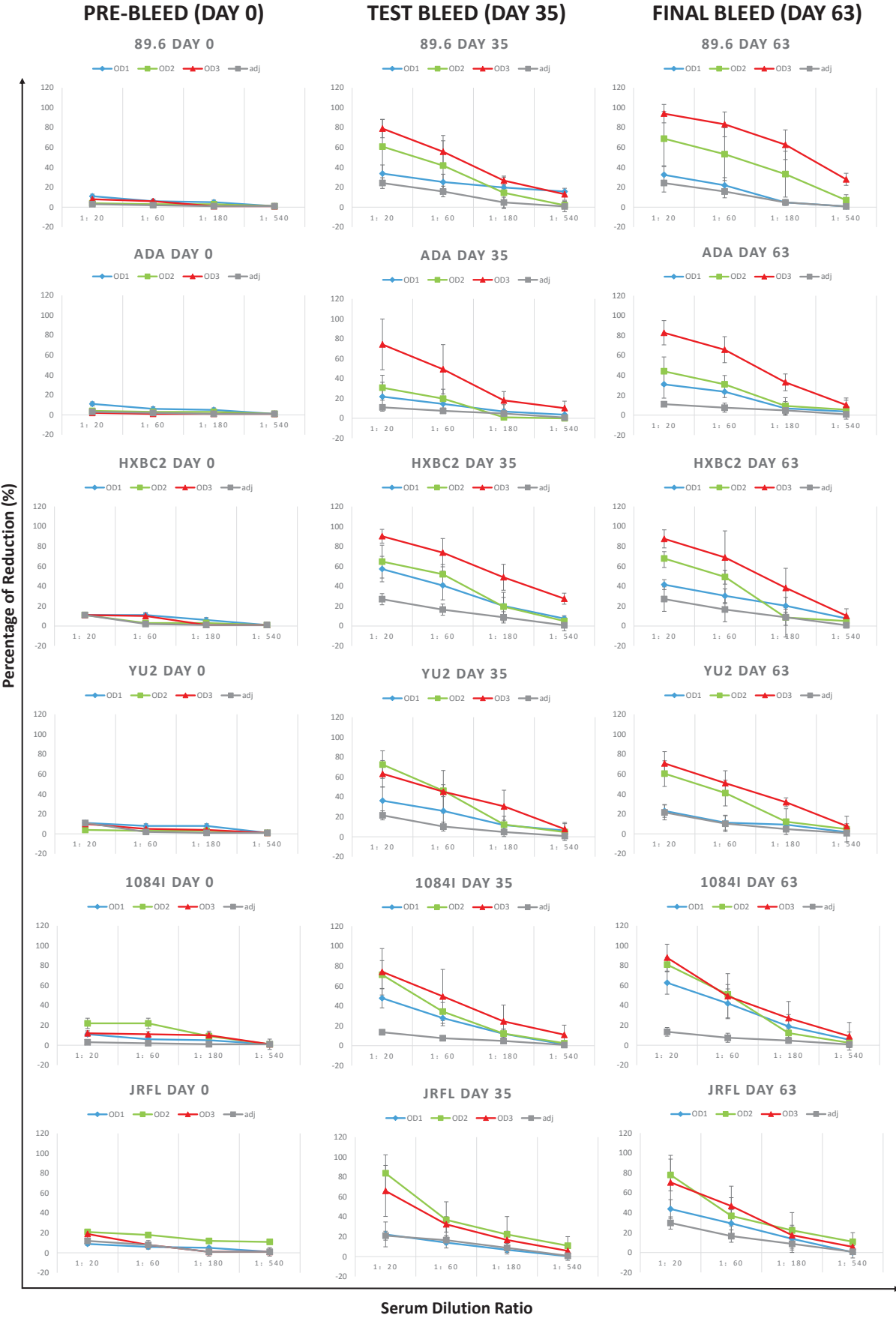

Supplement: Supplementary data 1 [file mmc1.pdf]
